# Supplementary material for: Resolving the full spectrum of human genome variation using Linked-Reads
Source: Genome Res. 2019 Apr;29(4):635–45. doi: 10.1101/gr.234443.118 (PMC6442396; doi:10.1101/gr.234443.118)
Supplement: Supplemental Material [file supp_gr.234443.118_Supplemental_longranger-2.2.zip › longranger-2.2.tar.bz2!/lr2.2-public/lib/stan/cmdstan/stan/doc/api/html/agrad_2rev_2functions_2is__nan_8hpp_source.html]

Stan: src/stan/agrad/rev/functions/is\_nan.hpp Source File


|  |  |
| --- | --- |
|  | Stan  2.5.0  probability, sampling & optimization |


- Main Page
- Related Pages
- Namespaces
- Classes
- Files

- File List
- File Members

All Classes Namespaces Files Functions Variables Typedefs Enumerations Enumerator Friends Macros Pages

- src
- stan
- agrad
- rev
- functions

is\_nan.hpp

Go to the documentation of this file.

1 #ifndef STAN\_AGRAD\_REV\_FUNCTIONS\_IS\_NAN\_HPP

2 #define STAN\_AGRAD\_REV\_FUNCTIONS\_IS\_NAN\_HPP

3

4 #include <stan/agrad/rev/var.hpp>

5 #include <stan/agrad/rev/internal/v\_vari.hpp>

6 #include <stan/math/functions/is\_nan.hpp>

7 #include <stan/math/constants.hpp>

8

9 namespace stan {

10

11  namespace agrad {

12

21  inline

22  int

23  is\_nan(const var& v) {

24  return stan::math::is\_nan(v.val());

25  }

26

27  }

28 }

29

30 #endif

stan::agrad::is\_nan

int is\_nan(const fvar< T > &x)

Returns 1 if the input's value is NaN and 0 otherwise.

**Definition:** is\_nan.hpp:22

stan::agrad::var::val

double val() const

Return the value of this variable.

**Definition:** var.hpp:209

v\_vari.hpp

var.hpp

stan::math::is\_nan

int is\_nan(const double x)

Returns 1 if the input is NaN and 0 otherwise.

**Definition:** is\_nan.hpp:19

stan::agrad::var

Independent (input) and dependent (output) variables for gradients.

**Definition:** var.hpp:27

is\_nan.hpp

constants.hpp


---

[ Stan Home Page ]

*© 2011–2014,
Stan Development Team.*
